# An Ethical Analysis of Public Attitudes towards Controlled Human Infection Studies in Singapore: Acceptability and Payment

**DOI:** 10.1007/s41649-024-00335-z

**Published:** 2025-05-30

**Authors:** Barnaby Young, Alberto Giubilini, Xin Hui Sam, Tamra Lysaght, Paul Anantharajah Tambyah, G Owen Schaefer, Julian Savulescu

**Affiliations:** 1 National Centre for Infectious Diseases, Singapore; 2 Department of Infectious Diseases, Tan Tock Seng Hospital, Singapore; 3 Lee Kong Chian School of Medicine, Nanyang Technological University Singapore, Singapore; 4 Uehiro Oxford Institute for Practical Ethics, University of Oxford, Oxford, UK; 5 Sydney School of Public Health, The University of Sydney, Faculty of Medicine and Health, Sydney, NSW Australia; 6 Centre for Biomedical Ethics, Yong Loo Lin School of Medicine, National University of Singapore, Singapore; 7 Division of Infectious Diseases, Department of Medicine, National University Hospital, National University Health System, Singapore; 8 Yong Loo Lin School of Medicine, National University of Singapore, Singapore; 9 Infectious Diseases Translational Research Programme, Department of Medicine, Yong Loo Lin School of Medicine, National University of Singapore, Singapore; 10 Biomedical Research Group, Murdoch Children’s Research Institute, Melbourne, VIC Australia

**Keywords:** Controlled human infection model, Coronavirus, Human challenge, Payment, Survey

## Abstract

Singapore is conducting its first controlled human infection (CHI) study, and is administering SARS-CoV-2 as the challenge agent. Ahead of this study, we conducted a survey to assess public perceptions in Singapore of CHI studies in general and with SARS-CoV-2, and the ethical issues they raise, including those around payments to research participants. Overall, there was large support for challenge studies in Singapore, suggesting they could obtain a social license. However, a minority strongly disagreed, and most respondents reported limited pre-survey knowledge about CHI studies. Importantly, Singaporeans support a higher incentive model of payment than is usually employed in challenge study research. They support either a Market Model or a Payment for Risk Model. There was most support for paying participants the highest rate offered—in our study, it was $SGD30 per hour. These results were broadly in line with a similar study in the UK, despite the latter having notably lower reported levels of public trust and, most recently, a highly criticized response to COVID-19. As such, general support for CHI studies may not be a direct function of background confidence in public or biomedical institutions but reflect other factors such as their intrinsic value and importance. More direct cross-cultural research in different contexts concerning attitudes towards CHI studies could help shed light on the extent that localized factors such as culture, history, and infrastructure affect both their acceptability and attitudes towards participant payment.

**Supplementary Information:**

The online version contains supplementary material available at 10.1007/s41649-024-00335-z.

## Introduction

Controlled human infection (CHI) studies are a clinical research study design involving the purposeful inoculation of volunteers with an infectious agent such as a virus or bacteria. Such studies are done to test the efficacy of medical interventions in a controlled setting and to study the pathogenesis of an infection. Field studies of “natural” infection are inherently limited by uncertainties in infection exposure and its detection, which is overcome in CHI (also known as human challenge) studies by directly engineering the exposure. Such studies began in 1796 when Edward Jenner inoculated 8-year-old James Phipps with cowpox, and, after subsequently exposing Phipps to smallpox, demonstrated that this cowpox vaccine protected against smallpox infection (Riedel 2005).

CHI studies are generally quicker to conduct and more cost-effective than the traditional sequential phase I–II randomized clinical trial design, as they can produce high-quality data from a smaller number of research participants. This feature makes them a particularly valuable experimental design in a public health emergency, where accelerating the development of vaccines is expected to translate into more lives saved (Watson et al. 2022). High impact breakthroughs made possible by challenge studies include a v-tetanus vaccine (Jin et al. 2017), proof of efficacy of an oral cholera vaccine (Tacket et al. 1999), and the malaria RTS,S vaccine (Moon et al. 2020).

However, because they involve the purposeful infection of volunteers, CHI studies also raise ethical questions as to when this act itself, and the risks it involves, can be justified by the potential benefits (e.g. Bambery et al. 2016; Hope and McMillan 2004). The growing acceptability of challenge studies among researchers, the voluntary nature of participation, and the presence of rigorous oversight by Institutional Review Boards (IRBs) mitigate concerns (Adams-Phipps et al. 2023). However, questions about what counts as an acceptable level or risk and the optimal way of obtaining valid, informed consent contribute to their ethical complexity.

Issues surrounding consent are also raised with the approach to compensation or payment of research participants. Some have suggested that the risks involved in CHI studies should be reflected in payment as a matter of fairness and to avoid exploitation of participants (Anomaly and Savulescu 2019; Grimwade et al. 2020). Others are concerned that payments for risks, or indeed payments for enrolment in challenge studies beyond reimbursement of reasonable costs, might be a form of undue inducement (Jefferson 2005), undermining informed consent or even potentially representing a form of coercion.

Singapore has initiated its first CHI study. This study administers SARS-CoV-2 as the challenge agent and is expected to lead to a clinical trial of a candidate transmission blocking vaccine as part of the Mucosal Immunity in Human Coronavirus Challenge (MusICC) consortium. All such studies will need to meet the local regulatory requirements, but beyond legal permissibility it is also important for major new biomedical initiatives to secure what has been termed a “social license”. This social license refers to a given practice or intervention being broadly acceptable to the public, in line with society’s expectations, values, and priorities (Stronge et al. 2024). The social license helps underpin the legitimacy of a practice (Demuijnck and Fasterling 2016), and in this case, securing and maintaining the social license can minimize risks of a public backlash as CHI studies proceed (Carter et al. 2015)..

As such, it is important to assess public awareness, understanding, and perceptions of challenge trials in Singapore generally and with SARS-CoV-2 specifically, as well as the ethical issues they raise, including about possible payments for research participants. We aim to assess these perceptions using survey methodology complementary to those carried out in the UK. These studies assessed British public perceptions of the acceptability of a SARS-CoV-2 challenge study (Barker et al. 2022) and the ethical permissibility of challenge studies on the basis of different levels of risk involved with different models of compensation to research participants (Grimwade et al. 2020).

Beyond assessing acceptability in Singapore, our study also aimed to shed some light on cross-cultural variation (or lack thereof) concerning attitudes towards CHI studies. Singapore is a reportedly high-trust, racially, and religiously diverse society which has made significant public and private investment in biomedicine (Noor and Leong 2013; Tan and Tambyah 2011). Previous research has found substantial levels of trust in government agencies that extends to public management of biomedical data (Lysaght et al. 2021). The present study explores whether we might expect the same level of trust to extend to a CHI study.

## Methods

For comparability of results, the survey administered replicated previously published studies conducted in the UK with light edits to reflect Singapore’s context and the evolving pandemic (Barker et al. 2022; Grimwade et al. 2020). There were three parts to the survey: (1) public awareness and perspective on CHI studies in general, and personal feelings about participating in a CHI study with SARS-CoV-2; (2) public practices and attitudes towards reimbursement in CHI studies; and (3) demographic data of survey respondents.

After an introductory question to ask respondents if they were aware of CHI studies, respondents viewed a short amination that explained the concept of a CHI study using coronavirus as an example. Hypothetical scenarios and statements relating to CHI studies and payment practices were utilized to assess the attitudes towards the study conduct and reimbursement for volunteers in the local context. See supplemental material for the full survey.

Respondents were recruited from the Health Opinions Panel Singapore (HOPS) and participants attending the research clinic at the National Centre for Infectious Disease. HOPS is an online research panel operated by the Centre for Biomedical Ethics, National University of Singapore, and was primarily recruited from an existing database at the Saw Swee Hock School of Public Health. The panel was selected from a sampling frame of de-identified household addresses provided by the Singapore Department of Statistics (SingStat) as a nationally representative sample.

The survey was disseminated using the REDCap online survey tool to the 2515 members of the HOPS panel in May 2023, and the identical survey was also administered to volunteers at NCID between February–August 2023. All respondents were reimbursed $20 for their time (estimated at 20–30 min) upon completion of survey.

The questionnaire was anonymous and self-administered. Responses were collected using single- or multiple-choice format with pre-defined options, 7-point Likert scale, and free-text comments to a series of questions and statements. Survey results were analyzed collectively, and then compared between the HOPS and NCID enrolled cohorts.

Descriptive statistics of the survey results were analyzed and compared between groups using the chi-square test for categorical variables, Student’s *t*-test for continuous as appropriate. For survey questions where respondents were asked for their agreement with statement on a 7-point Likert scale, summary data is presented in the text simplifying to a 3-point scale (agree/neutral/disagree).

This study was performed in line with the principles of the Declaration of Helsinki. Approval for this study was granted by the Ethics Committee of the National Centre for Infectious Diseases, the Domain Specific Review Board (Ref DSRB 2022/00845). Informed consent was obtained from all individual participants included in the study.

## Results

A total of 612 responses were collected in this study, of which 532 were from the HOPS cohort and 80 from NCID. There were no incomplete survey responses. Respondents’ characteristics are presented in Table 1 and were similar between the two cohorts (Supplementary Table 1).

**Table 1:** Summary demographic characteristics of survey respondents

Characteristics		*n*	%	Characteristics		*n*	%
Sex				Employment status			
	Female	343	56.0		Full-time	360	58.8
	Male	269	44.0		Part-time	44	7.2
					Self-employed	51	8.3
Age group					Student	11	1.8
	21–30	76	12.4		Retired	78	12.7
	31–40	164	26.8		Unemployed	28	4.6
	41–50	140	22.9		Homemaker	34	5.6
	51–60	94	15.4		Other	6	1.0
	61–70	99	16.2				
	> 70	39	6.4	Healthcare worker			
					Yes	76	12.4
Residential status					No	536	87.6
	Citizen	560	91.5				
	Permanent resident	47	7.7	Marital status			
	Employment/work pass	5	0.8		Single	395	64.6
					Married	178	29.1
Ethnicity					Divorced	35	5.7
	Chinese	507	82.8		Widowed	4	0.7
	Malay	35	5.7				
	Indian	51	8.3	Annual income (‘000)			
	Others	19	3.1		< 20	103	16.8
					20–60	186	30.4
Educational level					60–100	131	21.4
	No formal	1	0.2		> 100	63	10.3
	Primary school	7	1.1		Prefer not to say	129	21.1
	Secondary school	83	13.6				
	Post-secondary	34	5.6	Caring responsibility			
	Diploma	129	21.1		Yes	334	54.6
	Degree/post-graduate	358	58.5		No	278	45.4

The study population consisted of 269 (44%) males with a median age of 45 years (interquartile range [IQR] 34–59). The respondents predominantly identified as ethnic Chinese (507; 83%), Indian (51; 8%), or Malay (35; 6%), and 607 (99%) were local residents (citizens or permanent residents) and 358 (59%) educated to university degree or above. Full-time employment was reported by 360 respondents (59%) and the modal annual income was between S$20,000 and S$60,000 (30%). Only a small proportion of participants reported they were an employer or line manager (10%), and so this section of the survey was not analyzed further.

### Public’s Knowledge and Acceptability of CHI Studies in Singapore

Overall, 494 (81%) of the survey respondents either had no prior knowledge of CHI studies before taking part in the survey or were not sure if they did. Of the 118 (19%) respondents who reported prior knowledge, main sources were either news/media coverage 46 (39%) or social media 39 (33%). After watching the animation, 373 (61%) agreed that a CHI study with SARS-CoV-2 should take place in Singapore and 85 (14%) disagreed, while 154 (25%) were neutral.

### Potential Problems for Volunteers to be Isolated for 2 weeks

Respondents were questioned on the challenges they feel volunteers may face when participating in a CHI study which involves quarantine at a specialized medical isolation unit for 2 weeks. The potential negative impact on physical health (such as short- and long-term symptoms of coronavirus infection) and mental health (such as getting bored, stressed, lonely, anxious, angry) were listed by more than half of the respondents, 341 (56%) and 330 (54%) respectively, as the top potential problems. Issues concerning the duration of the study including separation from household members 283 (46%), inability to carry on working 273 (45%), and consumption of annual leave 272 (44%) were identified as other major concerns.

### Opinion Statements relating to a Human Challenge Study with Coronavirus

**Fig. 1:**
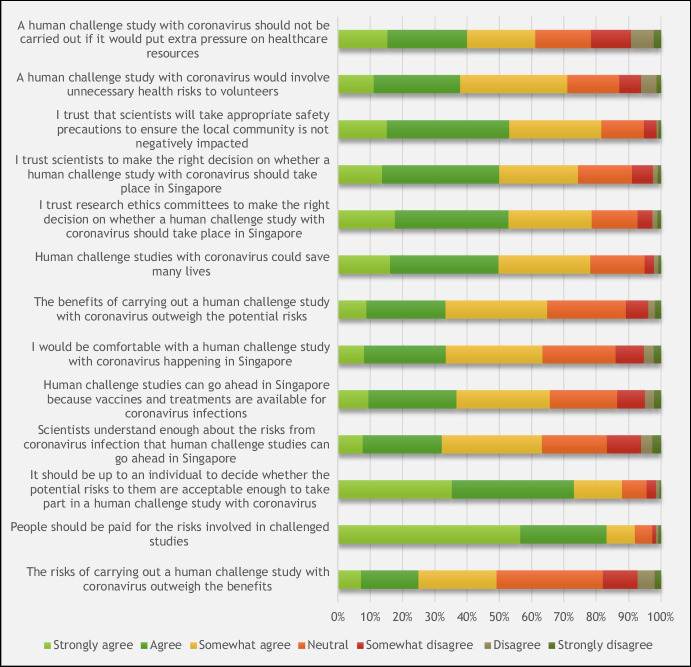
Summary of respondent agreement with statements on human challenge studies with coronavirus, on a 7-point Likert scale (*n* = 612)

There were 301 (49%) respondents who agreed that “The benefits of carrying out a human challenge study with coronavirus outweigh the potential risks” (Fig. 1). A further 201 (33%) were neutral and 110 (18%) disagreed. Most respondents agreed that “Human challenge studies with coronavirus could save many lives” (479, 78%).

A high degree of trust in Institutional Review Boards was reported, with 481 (79%) agreeing that “I trust research ethics committees to make the right decision on whether a human challenge study with coronavirus should take place in Singapore”. Similarly, 499 (82%) of respondents indicated agreement with their trust in scientist: “I trust scientists will take appropriate safety precautions to ensure the local community is not negatively impacted”. Their trust in scientists was also reflected in agreement to the statements that “I trust scientists to make the right decision on whether a human challenge study with coronavirus should take place in Singapore” (455, 74%). The surveyed respondents believed it is “up to an individual to decide whether the potential risks to them are acceptable enough to take part in a CHI study with coronavirus” (538, 88%) and also that volunteers “should be paid for the risks involved” (563, 92%).

### Personal Feelings about Participating in a Human Challenge Study with Coronavirus

**Fig. 2:**
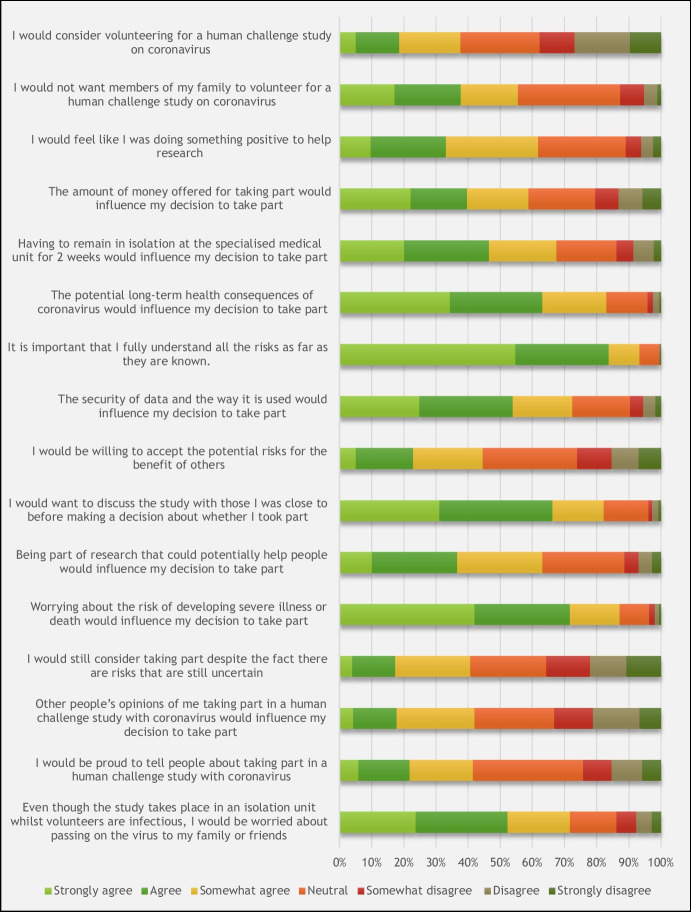
Summary of respondent agreement with statements relating to their participation in a human challenge study with coronavirus, on a 7-point Likert scale (*n* = 612)

At a personal level, only 230 (38%) would consider volunteering for a CHI study with SARS-CoV-2 and 340 (56%) would not want their family members to volunteer (Fig. 2).

Respondents reported that participating in a CHI study would make them feel like they were doing something positive to help research (378, 62%) and could potentially help people (386, 63%). Most people would want to discuss participating in a CHI study with their close ones (503, 82%), but would worry about passing on the virus to their family and friends (439, 72%). Respondents generally agreed that it is important to fully understand the risks of participation as far as they are known in order to make an informed and considered decision (571, 93%). In comparison, relatively fewer respondents (378, 62%) felt that the amount of money offered for taking part would influence their decision to take part.

### Practices and Attitudes towards Payment in Challenge Studies

Two hypothetical CHI scenarios were shared with participants: the first was a study of an attenuated dengue virus as the challenge agent conducted in an outpatient setting; the second a challenge study with SARS-CoV-2 in which volunteers must stay in isolation for at least 2 weeks. In both studies, the risk of serious adverse events from the infection was described as unlikely. A total of 486 (79%) and 492 (80%) of respondents believed people should be allowed to participate in the CHI studies outlined in scenarios 1 and 2, respectively.

The National Wages Council (NWC) in Singapore had recommended a basic wage threshold of approximately S$8 per hour in their guidelines issued in May 2021 (National Wages Council 2021). The median gross income of a full-time employed Singapore resident in the same year was reported to be approximately S$25/h. Among those individuals who responded that they agreed with the CHI study going ahead, for both scenarios, the most widely chosen amount per hour for reimbursement was S$30 (232, 48% and 235, 48%), followed by S$25–30 (102, 21% and 89, 18%) and S$20–25 (65, 13% and 55, 11%). It is worth noting that only ~ 2% of respondents indicated no payment should be made to volunteers.

There was a high degree of concordance between the responses submitted by participants for each of the two scenarios. For the 404 (66%) of participants who disclosed their annual income, the proportion of participants who selected the maximum reimbursement amount (S$30) increased with income—from 42% among those with annual income < S$20,000 per year to 73% with annual income > S$100,000.

### Determination of Reimbursement for CHI Study Volunteers

**Fig. 3:**
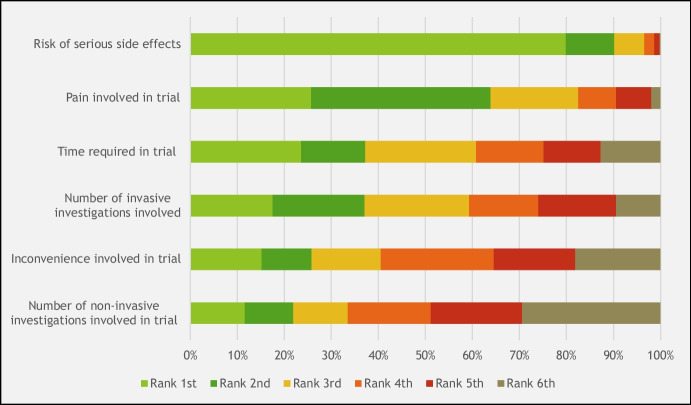
Summary of respondent agreement with statement on what factors they believe should be taken into account when determining payment for challenge study participants, on a 7-point Likert scale (*n* = 612)

Respondents were asked to rank the importance of various factors in determining payment amounts (Fig. 3). Overall, 454 (74%) felt that all the factors mentioned, namely risk of serious side effects, pain involved, time required, number of invasive and non-invasive investigations, and inconvenience cause by the trial, should be considered when deciding on appropriate compensation. Among these, the risk of serious side effects was selected by 489 (80%) of the sample population as the most important factor.

**Fig. 4:**
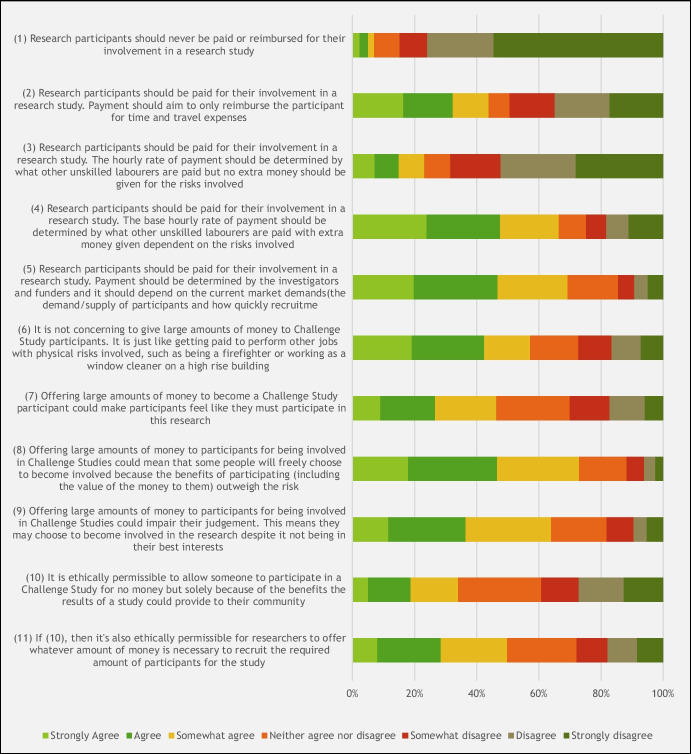
Summary of respondent agreement with statements about how the amount of payment should be determined in challenge studies, on a 7-point Likert scale (*n* = 612)

In terms of payment structure, the highest level of agreement (424 [69%]) was for the statement “payment should be determined by the investigators and funders and it should depend on the current market demand (the demand/supply of participants and how quickly recruitment needs to occur)”—the “Market Model” for payment (Fig. 4). This was followed by “the base hourly rate of payment should be determined by what other unskilled labourers are paid with extra money given dependent on the risks involved”—the “Payment for Risk Model” (407, [67%]).

Despite the general agreement that CHI participants should get paid, 391 (64%) of respondents agreed that “Offering large amounts of money to participants for being involved in Challenge Studies could impair their judgment. This means they may choose to become involved in the research despite it not being in their best interests”. Conversely, however, it was also acknowledged that “Offering large amounts of money to participants for being involved in Challenge Studies could mean that some people will freely choose to become involved because the benefits of participating (including the value of the money to them) outweigh the risks” (447, 73%).

### Effects of Prior Experience as a Research Participant

Previous experience as a research participant was reported by 187 (31%) of survey respondents (see Supplementary Table 2). Prior knowledge about CHI studies was similar between both groups (34 (18%) with experience vs 84 (20%) without; *p* = 0.60). However, respondents with prior experience of research participation reported greater acceptability of CHI studies (131 (70%) vs 242 (57%); *p* = 0.0022), and also greater willingness to participate (93 (50%) vs 137 (32%); *p* < 0.001).

## Discussion and Ethical Analysis

There are several ethically relevant insights that emerge from this survey. The large support for challenge studies from this cross section of the public provides support for introducing them in Singapore. Medical research has a notoriously troubled history with human experimentation, including in Singapore, and implementing a policy of purposefully infecting people with a virus to test a novel vaccine without public support might have detrimental effects not only in terms of support of vaccination policies of public health importance, but of trust in science more generally (Abdul Majid 2021). After all, medical research depends on people’s trust for its funding and success (Wilson and Hunter 2010). This study suggests that CHI studies in Singapore will be able to obtain social license.

One major limitation of this survey is that despite engagement in other forms of research by many individuals, most respondents reported limited pre-survey knowledge about CHI studies. This emphasizes the need for public education and engagement during the launch of the CHI program. However, even with public education, it would be useful to understand why a small but significant minority disagreed with them. This may best be done with qualitative studies such as focus group discussions where time is provided to share detailed information about CHI studies and the nuances of participant responses can be understood.

While the majority supported the concept of CHI studies (almost two-thirds of respondents), only a minority would be prepared to enroll in a SARS-CoV-2 challenge study themselves (less than one-third) or would be supportive if one of their family members were to enroll. Even among those with previous experience as research participants, the majority—although a small one—said they would not be willing to volunteer as participants.

This discrepancy might reflect the population surveyed where most would not meet eligibility criteria for typical CHI studies due to age, medical conditions, or other study requirements. The Barker survey was conducted in October 2020 during the height of the public health emergency prior to vaccines being available, and with many COVID-19-related restrictions in place. This context may explain why more than 60% of respondents in the UK would be supportive of an immediate family member participating in a SARS-CoV-2 challenge study, compared with only 38% in Singapore. It might also contribute to the high percentage of support for CHI in Singapore in 2023 as most of the population had been either infected or vaccinated or both by then. Decision-making on participation in research studies has been observed to be different in parts of Asia compared with Western countries, with a greater involvement of family members in this process in Asia (Susilo et al. 2019). Whether this applies to potential CHI study participants in Singapore is not known, but the survey highlights the importance of a carefully managed informed consent process, including providing ample time, information, and privacy for potential participants to consider and discuss their participation with people close to them, and ensuring consent is treated as a continuous, dynamic process.

That most survey respondents would not want to participate in a challenge study is not surprising. The tension between third-person, ethical judgments (“this is the right/a good thing to do”) and first person, self-interested judgments (“I am not going to do it myself/I am not willing to pay the cost of it myself”) is both a common feature of human thinking and something that has been observed with other proposed health policies. For instance, people’s views on antimicrobial prescribing, in light of the problem of antimicrobial resistance, present the same tension (Dao et al. 2019): when asked whether doctors should prioritize patients or public interest in antibiotic prescription, respondents largely sided with the public, but when asked the same question in the case they themselves were the patient, support for the society-first approach was significantly lower. This is similar to the “Not-in-my backyard” situation where people recognize the need for a common good but are not willing to pay any price to contribute to it themselves.

CHI studies present the same problem: they have the potential to benefit public health by delivering better interventions such as vaccines in a faster way. But there will be some risk and inconvenience to individuals who enroll in these studies for the sake of societal benefit. This raises two issues. One, strictly utilitarian in nature, is about creating enough incentives for enough individuals to enroll. The second, fairness-based, is about ensuring that those who do enroll are adequately compensated and not induced to participate against their best interests such as by concealing underlying medical conditions. The first issue does not seem to raise particular concerns: as said at the beginning, one advantage of challenge studies is that they require fewer participants than traditional trials. If one-third of the population was willing to enroll, even with strict study eligibility criteria for participation, enough people are likely to be identified to be able to run many successful challenge studies. However, the second issue is more complex, as is the question of what counts as fair treatment.

In our view, fair compensation for participating in a CHI study might justifiably take into account more than time and expenses—as is normally the case for traditional trials—but also the risks regarding participant physical and mental health. This is particularly the case when participants may not be expected to directly benefit from their participation (Lynch et al. 2021). This is a complex issue, but is arguably no different from what is already the case in other contexts, for example in the case of certain jobs, where the risks associated with that job are reflected in the remuneration.

What is striking in this study is that over 60% of Singaporeans supported either a Market Model or a Payment for Risk Model of payment. Neither of these represents the status quo when research ethics committees are assessing compensation to participants in clinical research, which is based on subjective estimates of compensation for time and burden (e.g. pain). This is interesting in and of itself as it might be an example of the more general discrepancy, observed elsewhere, between the ethical views of the public and those of bioethics expertise (Pierson et al. 2024).

Some would see payments for risks as a form of coercion or undue inducement—that is, as something that would circumvent people’s rational capacity for autonomous choice. A lot of these objections depend very much on what is meant by “coercion”. On some accounts of coercion (Millum and Garnett 2019), offers of payments and incentives could count as coercive, because they put excessive psychological pressure on individuals—making “offers that they cannot reasonably refuse”. Thus, a payment for risks would add monetary value to enrolment in challenge studies, which would make it too tempting for some people. Other accounts of coercion (Wertheimer and Miller 2008), however, argue that only threats of penalties can meaningfully coerce, as they restrict the range of options open to a person. For instance, the paradigmatic case of “your money or your life” is a case of coercion because I cannot keep both my money and my life, so my range of options compared to the relevant baseline is restricted. These divergent accounts of coercion, though, share a common ethical thread: certain actions can significantly undermine autonomy and rational capacity. As such, whatever one’s view of coercion, what matters is whether paying people for risks, or indeed paying them higher sums than what is normally done in the case of traditional trials, significantly undermines their autonomy and rational capacity.

Again, this largely comes down to how autonomy and rationality are defined, which is a complex philosophical question beyond the scope of this paper, and to empirical evidence about how monetary incentives affect participants’ decision-making—for instance, evidence suggesting limited influence of monetary incentives on decisions to enroll in studies (Largent et al. 2022) would count against the claim that autonomy and rational capacity are undermined. For the purpose of this paper, we are examining whether any perceived limitation of autonomy would be considered acceptable by the society implementing that policy. The general support in our sample for market- or risk-based payment is indeed consistent with other generally socially acceptable practices around compensation, even if there is some tradeoff in terms of autonomy. Here is why.

Let’s assume, for the sake of argument, that higher payments for risk in some sense limit autonomy in deciding whether to enroll (Savulescu 2001). This would entail that any additional payments for the risks associated with more hazardous occupations—such as special military duties or firefighting—also limit people’s autonomy to take up those jobs. But we normally do not think in those terms in the case of hazardous occupations, so why then should it be a reason for preventing people from accepting larger payments for risks and other costs in challenge trials? To say that payments undermine autonomy in one case, but not the other, is inconsistent. Some have further suggested that preventing people from enrolling in paid research out of concerns for undue inducement would be a form of unjustifiable paternalism (Savulescu 2001), which is normally not adopted in the case of other choices made for the sake of money. To the extent that payments undermine autonomy, they do it across the board, whether we are talking of medical research or of the choice of hazardous occupations.

Some would appeal to “research exceptionalism” (Wilson and Hunter 2010), that is, the idea that medical research is somehow special and different from other contexts, such that ethical considerations that apply elsewhere are not straightforwardly transferrable to research. Perhaps medical research is different and ethical constraints apply that would not apply to, say, the case of choosing highly paid, hazardous occupations. Research exceptionalism—assuming it is justified—is based in part on the idea mentioned earlier that research depends on public trust and obtaining a social license, and that public trust could be undermined in a context where those who take on themselves the risks do not receive any significant benefit (Wilson and Hunter 2010). However, public support for a CHI study would significantly weaken the case for research exceptionalism in this context, as it seems plausible to suppose that trust would not be undermined by practices enjoying social license.

In this respect, our study clearly suggests that a significant majority of the Singapore population (around 80%) would not only allow participants to enroll in challenge studies with varying levels of risk, but also that risk of serious side effects should be factored in when determining the level of payment for participants, alongside consideration of pain involved, time required, number of invasive and non-invasive investigations, and inconvenience. It would be difficult on this basis to suggest that payment for risks in challenge trials would undermine public trust, since it is precisely what the public would support. Interestingly, this is consistent with the finding by Grimwade and colleagues (2020) among the UK public, suggesting that objections to CHI studies on the grounds of excessive payments and undue inducement may be out of touch with public values. Moreover, among both Singaporean and UK respondents, the Market Model and the Risk Model of payment—the former based on supply/demand of participants and the latter on the level of risk involved—enjoy wide public support (with Grimwade et al.’s study showing slightly larger support for the Payment for Risk over the Market Model).

Consistently with the ethical analysis carried out in that study, it is worth pointing out that an ethically relevant consideration is the risk of exploitation. While some are concerned about too high payments as a possible threat to autonomy—a concern we have suggested is not well founded—there are risks with paying people too little, too. Too small payments might result in exploitation of their labour and good will to contribute to valuable research or of their decision to enroll in the study because of the need for money. Thus, one increased benefit of larger payments for participation in research trials is that it could prevent exploitation (Anomaly and Savulescu 2019), a point which seems further supported by this study’s findings on Singapore’s positive public attitudes towards payments for risks, and the observed correlation between expected reimbursement and income. The counter argument to this is commonly expressed in Singapore where “local” Singaporeans are not willing to do dangerous and risky jobs such as construction or undersea ship repair so they are outsourced to migrant workers who come from low-income countries and are prepared to take risks for salaries which would not attract Singaporeans. These workers are recognized by local institutional review boards as vulnerable populations and the same arguments would likely be applied to participants in CHI studies to ensure that they are protected under standard guidelines.

This survey has several limitations. A relatively large number of respondents completed the survey and across a wide range of ages and ethnicities, however, this sample was self-selected, and hence biased towards individuals with an interest in medical research, including a substantial proportion who had experience as participants. The survey was conducted solely in English and individuals who were not sufficiently fluent in this language were excluded from participation. Further studies are required to determine if the results of the survey are representative of the general population. Respondents may have interpreted some of the questions or their response selection differently to the survey writer’s intention, and as a self-directed questionnaire, participants did not have the opportunity to confirm their understanding. The order of questions may also have affected responses, though given the detailed set of questions respondents completed, we do not believe the magnitude of this effect would have been large. At the time of the survey in February 2023, the last COVID-19 pandemic public health measures in Singapore were lifted; movement and contact limitations had been removed in Singapore less than a year previously. While fear and anxiety related to COVID-19 remain elevated, the urgency for development of new vaccines and therapeutics has waned (Broockman et al. 2021). Attitudes towards CHI studies with SARS-CoV-2 are likely to have evolved over the course of the pandemic along with the judgment of individual risks from participation vs societal benefits (Holm 2020). Whether that will have changed the publics conception of their overall acceptability is not known.

## Conclusions

In light of these findings, it is likely that the Singaporean population would not have serious concerns with or objections to the prospect of CHI studies run under appropriate regulation and supervision. Existing safeguards should be sufficient to secure the social license. These results are perhaps not too surprising given, as noted above, Singaporeans generally put high levels of trust into public institutions including in the biomedical sphere. And that trust is arguably earned—for example, Singapore is noted for having one of the most successful approaches to the COVID-19 pandemic through stringent but effective governance and public health management mechanisms (Chua et al. 2020; Kuguyo et al. 2020; Woo 2020). The main caveat to this is the lack of knowledge of CHI even by individuals who had participated in biomedical research previously. This emphasizes the importance of public education similar to what has been done to address vaccine hesitancy in the country.

Importantly, Singaporeans are likely to support a higher incentive model of payment than is usually employed in challenge study research. They support either a Market Model or a Payment for Risk Model. There was most support for paying participants the highest rate offered—in our study, this was SGD 30 per hour.

Notably, while we found similar support for CHI in Singapore as in the UK, a country with a reportedly lower level of trust in healthcare, we found a much lower willingness to participate in such studies themselves and a greater demand for market level compensation. This may reflect the differences in Singapore’s hybrid healthcare financing system which mandates user fees compared to the UK’s national health system. Given that people in Singapore are conditioned to expect to pay market rates for their healthcare (with subsidies for most of the population), it is quite understandable for them to want to be compensated at market rates for participation in CHI studies. More direct cross-cultural research in different contexts concerning attitudes towards CHI studies could help shed light on the extent that localized factors such as culture, history, and healthcare infrastructure might impact the possibility of them obtaining a social license.

## Supplementary Information

Below is the link to the electronic supplementary material.

Supplementary file1 (DOCX 288 KB)

## Data Availability

Anonymised data available on request to the corresponding author.
