# Supplementary material for: An Ethical Analysis of Public Attitudes towards Controlled Human Infection Studies in Singapore: Acceptability and Payment
Source: Asian Bioeth Rev. 2025 May 30;18(1):117–34. doi: 10.1007/s41649-024-00335-z (PMC12824035; doi:10.1007/s41649-024-00335-z)
Supplement: Supplementary file 1 — Supplementary file1 (DOCX 288 KB) [file 41649_2024_335_MOESM1_ESM.docx]

**Supplementary material**

Supplemental Table 1: Characteristics of HOPS and NCID Respondents

| **Characteristics** | | **HOPS** | | **NCID** | | | | |
| --- | --- | --- | --- | --- | --- | --- | --- | --- |
|  |  | **n** | **%** | **n** | | **%** | |  |
| Sex |  |  |  |  | |  | |  |
|  | Female | 299 | 56.2 | 44 | | 55.0 | |  |
|  | Male | 233 | 43.8 | 36 | | 45.0 | |  |
| Age group |  |  |  |  | |  | |  |
|  | 21-30 | 50 | 9.4 | 26 | | 32.6 | |  |
|  | 31-40 | 131 | 24.6 | 33 | | 41.3 | |  |
|  | 41-50 | 128 | 24.1 | 12 | | 15.0 | |  |
|  | 51-60 | 91 | 17.1 | 3 | | 3.8 | |  |
|  | 61-70 | 96 | 18.1 | 3 | | 3.8 | |  |
|  | >70 | 36 | 6.8 | 3 | | 3.8 | |  |
|  |  |  |  |  | |  | |  |
| Residential status | |  |  |  | |  | |  |
|  | Citizen | 497 | 93.4 | 63 | | 78.8 | |  |
|  | Permanent resident | 35 | 6.6 | 12 | | 15.0 | |  |
|  | Employment/Work Pass | 0 | 0.0 | 5 | | 6.3 | |  |
| Ethnicity |  |  |  |  | |  | |  |
|  | Chinese | 444 | 83.5 | 63 | | 78.8 | |  |
|  | Malay | 29 | 5.5 | 6 | | 7.5 | |  |
|  | Indian | 45 | 8.5 | 6 | | 7.5 | |  |
|  | Others | 14 | 2.6 | 5 | | 6.3 | |  |
| Educational level | |  |  |  | |  | |  |
|  | No formal | 1 | 0.2 | 0 | | 0.0 | |  |
|  | Primary school | 7 | 1.3 | 0 | | 0.0 | |  |
|  | Secondary school | 76 | 14.3 | 7 | | 8.8 | |  |
|  | Post-secondary | 32 | 6.0 | 2 | | 2.5 | |  |
|  | Diploma | 114 | 21.4 | 15 | | 18.8 | |  |
|  | Degree/Post-graduate | 302 | 56.8 | 56 | | 70.0 | |  |
| Employment status |  |  |  |  | |  | |  |
|  | Full-time | 301 | 56.6 | 59 | | 73.8 | |  |
|  | Part-time | 40 | 7.5 | 4 | | 5.0 | |  |
|  | Self-employed | 47 | 8.8 | 4 | | 5.0 | |  |
|  | Student | 5 | 0.9 | 6 | | 7.5 | |  |
|  | Retired | 76 | 14.3 | 2 | | 2.5 | |  |
|  | Unemployed | 26 | 4.9 | 2 | | 2.5 | |  |
|  | Homemaker | 32 | 6.0 | 2 | | 2.5 | |  |
|  | Other | 5 | 0.9 | 1 | | 1.3 | |  |
| Healthcare worker |  |  |  |  | |  | |  |
|  | Yes | 39 | 7.3 | 37 | | 46.3 | |  |
|  | No | 493 | 92.7 | 43 | | 53.8 | |  |
| Marital status |  |  |  |  | |  | |  |
|  | Single | 145 | 27.3 | 44 | | 55.0 | |  |
|  | Married | 351 | 66.0 | 33 | | 41.2 | |  |
|  | Divorced | 33 | 6.2 | 2 | | 2.5 | |  |
|  | Widowed | 3 | 0.6 | 1 | | 1.3 | |  |
| Annual income (‘000) |  |  |  |  | |  | |  |
|  | <20 | 91 | 17.1 | 12 | | 15.0 | |  |
|  | 20-60 | 155 | 29.1 | 31 | | 38.8 | |  |
|  | 60-100 | 112 | 21.1 | 19 | | 23.8 | |  |
|  | >100 | 56 | 10.5 | 7 | | 8.8 | |  |
|  | Prefer not to say | 118 | 22.2 | 11 | | 13.8 | |  |
| Caring responsibility |  |  |  |  |  | |  |  |
|  | Yes | 302 | 56.8 | 32 | | 40.0 | |  |
|  | No | 230 | 43.2 | 48 | | 60.0 | |  |

Supplemental Table 2: Of the 612 respondents, 187 (30.6%) reported prior participation in health care research.

**Q: Have you read, seen or heard anything about the idea of a human challenge (also known as Controlled Human Infection) study?**

|  | Yes | No | Don’t Know |
| --- | --- | --- | --- |
| Overall (n=612) | 118 (19%) | 441 (72%) | 53 (9%) |
| With research experience (n=187) | 34 (18%) | 138 (74%) | 15 (8%) |
| Without research experience (n=425) | 84 (20%) | 303 (71%) | 38 (9%) |

**Q: Having watched the animation, to what extent do you agree or disagree that a human challenge study with coronavirus should take place in Singapore?**

|  | Strongly agree | Agree | Somewhat agree | Neutral | Somewhat disagree | Disagree | Strongly disagree |
| --- | --- | --- | --- | --- | --- | --- | --- |
| Without Research Experience (n=425) | 28 | 93 | 121 | 122 | 41 | 14 | 6 |
| Previous Research Participants (n=187) | 18 | 60 | 53 | 32 | 15 | 4 | 5 |
| Total (n=612) | 46 | 153 | 174 | 154 | 56 | 18 | 11 |

**Q: I would consider volunteering for a human challenge study on coronavirus**

|  | Total (n=612) | With research experience (n=187) | Without research experience (n=425) |
| --- | --- | --- | --- |
| Strongly Agree | 31 (5.1%) | 14 (7.5%) | 17 (4.0%) |
| Agree | 83 (13.6%) | 40 (21.4%) | 43 (10.1%) |
| Somewhat agree | 116 (19.0%) | 39 (20.9%) | 77 (18.1%) |
| Neutral | 15124.7%) | 42 (22.5%) | 109 (25.6%) |
| Somewhat disagree | 67 (10.9%) | 16 (8.6%) | 51 (12.0%) |
| Disagree | 105 (17.2%) | 19 (10.2%) | 86 (20.2%) |
| Strongly disagree | 59 (9.6%) | 17 (9.1%) | 42 (9.9%) |

*Willingness to participate in a CHI study was found to be higher among those with experience as research participants compared to those without (50% vs 32%, p<0.001).

**Exploring attitudes towards participation and payment in a Controlled Human Infection Model (CHIM) Study**

**Section A. Attitudes to human challenge with coronavirus in Singapore**

Before deciding to take part in this survey, had you read, seen or heard anything about the idea of a human challenge (also known as Controlled Human Infection) study?

1. Yes
2. No
3. Don't know

[If yes]

Where have you read, seen or heard about human challenge studies with coronavirus before? Please select all that apply.

1. News or media coverage
2. Social media
3. Personal conversation
4. In depth or scientific articles
5. Other - please specify:

To understand more about what a human challenge study involves and why they are carried out, please watch the following animation: https://www.youtube.com/watch?time_continue=1&v=FncT4ki-Uww&feature=emb_logo

Having watched the animation, to what extent do you agree or disagree that a human challenge study with coronavirus should take place in Singapore?

1. Strongly agree
2. Agree
3. Somewhat agree
4. Neutral
5. Somewhat disagree
6. Disagree
7. Strongly disagree

Taking part in a human challenge study with SARS-CoV-2, the coronavirus that causes COVID-19 would involve inpatient stay at a specialised medical isolation unit for approximately 2 weeks. Only young, healthy, vaccinated volunteers would be eligible to participate.

We would like to know your opinions about isolation at a specialised medical unit regardless of whether you were eligible and/or willing to take part in a study like this. Which, if any, of the following do you see as **potential** problems if you had to stay at a medical unit in isolation.

Please select **all** that apply. If your answer(s) do not appear in the list below, please type them into the 'Other' box provided. If you do not see any potential problems with staying at a medical unit in isolation, please select the 'Not applicable' option.

1. It might negatively affect my mental health (e.g. I would get bored, stressed, lonely, anxious, angry, etc.)
2. It might negatively affect my fitness (e.g. my nutrition would be different, I couldn’t exercise as usual, etc.)
3. It might negatively affect my physical health (e.g. I might experience short- or long-term symptoms of coronavirus)
4. I would find it difficult to separate myself from others in my household for 2 weeks (e.g. my partner, children, housemates, etc.)
5. I would find it difficult not being able to go outside.
6. I have caring responsibilities and would find it difficult to find someone to cover those (e.g. I care for a dependent child, someone with disabilities, an elderly relative, neighbour, etc.)
7. It would negatively affect my social life (e.g. it would negatively impact my friendships, I would miss attending social and cultural events, etc.)
8. I would not be able to carry on working
9. I would have to use my annual leave
10. I would experience a loss of income
11. My studies or education would suffer
12. My employer would not be understanding or supportive
13. Other [open] Please specify
14. Don't know
15. Not applicable - I do not see any potential problems from staying at a medical unit in isolation for a human challenge study

The following statements reflect different opinions on human challenge studies with coronavirus. Please indicate how much you agree or disagree with each statement below.

|  | Strongly agree | Agree | Somewhat agree | Neutral | Somewhat disagree | Disagre**e** | Strongly disagree |
| --- | --- | --- | --- | --- | --- | --- | --- |
| A human challenge study with coronavirus should not be carried out if it would put extra pressure on healthcare resources |  |  |  |  |  |  |  |
| A human challenge study with coronavirus would involve unnecessary health risks to volunteers |  |  |  |  |  |  |  |
| I trust that scientists will take appropriate safety precautions to ensure the local community is not negatively impacted |  |  |  |  |  |  |  |
| I trust scientists to make the right decision on whether a human challenge study with coronavirus should take place in Singapore |  |  |  |  |  |  |  |
| I trust research ethics committees to make the right decision on whether a human challenge study with coronavirus should take place in Singapore |  |  |  |  |  |  |  |
| Human challenge studies with coronavirus could save many lives |  |  |  |  |  |  |  |
| The benefits of carrying out a human challenge study with coronavirus outweigh the potential risks |  |  |  |  |  |  |  |
| I would be comfortable with a human challenge study with coronavirus happening in Singapore |  |  |  |  |  |  |  |
| Human challenge studies can go ahead in Singapore because vaccines and treatments are available for coronavirus infections |  |  |  |  |  |  |  |
| Scientists understand enough about the risks from coronavirus infection that human challenge studies can go ahead in Singapore |  |  |  |  |  |  |  |
| It should be up to an individual to decide whether the potential risks to them are acceptable enough to take part in a human challenge study with coronavirus |  |  |  |  |  |  |  |
| People should be paid for the risks involved in challenged studies |  |  |  |  |  |  |  |
| The risks of carrying out a human challenge study with coronavirus outweigh the benefits |  |  |  |  |  |  |  |

The following statements relate to **your personal feelings about participating** in a human challenge study with coronavirus. Please indicate how much you agree or disagree with each statement below, regardless of whether you think you would be eligible to take part in a human challenge study with coronavirus.

|  | Strongly agree | Agree | Somewhat agree | Neutral | Somewhat disagree | Disagree | Strongly disagree |
| --- | --- | --- | --- | --- | --- | --- | --- |
| I would consider volunteering for a human challenge study on coronavirus |  |  |  |  |  |  |  |
| I would not want members of my family to volunteer for a human challenge study on coronavirus |  |  |  |  |  |  |  |
| I would feel like I was doing something positive to help research |  |  |  |  |  |  |  |
| The amount of money offered for taking part would influence my decision to take part |  |  |  |  |  |  |  |
| Having to remain in isolation at the specialised medical unit for 2 weeks would influence my decision to take part |  |  |  |  |  |  |  |
| The potential long-term health consequences of coronavirus would influence my decision to take part |  |  |  |  |  |  |  |
| It is important that I fully understand all the risks as far as they are known. |  |  |  |  |  |  |  |
| The security of data and the way it is used would influence my decision to take part |  |  |  |  |  |  |  |
| I would be willing to accept the potential risks for the benefit of others |  |  |  |  |  |  |  |
| I would want to discuss the study with those I was close to before making a decision about whether I took part |  |  |  |  |  |  |  |
| Being part of research that could potentially help people would influence my decision to take part |  |  |  |  |  |  |  |
| Worrying about the risk of developing severe illness or death would influence my decision to take part |  |  |  |  |  |  |  |
| I would still consider taking part despite the fact there are risks that are still uncertain |  |  |  |  |  |  |  |
| Other people’s opinions of me taking part in a human challenge study with coronavirus would influence my decision to take part |  |  |  |  |  |  |  |
| I would be proud to tell people about taking part in a human challenge study with coronavirus |  |  |  |  |  |  |  |
| Even though the study takes place in an  isolation unit whilst volunteers are infectious, I would be worried about passing on the virus to my family or friends |  |  |  |  |  |  |  |

**[SCREEN_EMPLOYER]** Are you an employer or line manager?

1. Yes
2. No

[Display if **EMPLOYER = 1**]

The following statements relate to **an employer’s considerations about one of their employees participating** in a human challenge study with coronavirus. Please indicate how much you agree or disagree with each statement below. Same for this section

|  | Strongly agree | Agree | Somewhat agree | Neutral | Somewhat disagree | Disagree | Strongly disagree |
| --- | --- | --- | --- | --- | --- | --- | --- |
| I would support my employee as much as possible so they could take part |  |  |  |  |  |  |  |
| I am able to allow my employee to work flexibly so they can take part (e.g. remote working, reduced hours, etc) |  |  |  |  |  |  |  |
| I would be uncomfortable about the risk of harm to my employee |  |  |  |  |  |  |  |
| My employee would have to take time off work for the duration of the stay at the medical isolation unit in order to take part |  |  |  |  |  |  |  |
| I would be concerned my employee might need extended time off work due to longer term symptoms |  |  |  |  |  |  |  |
| I trust that there would not be an increased risk of transmission within my workplace due to my employee’s participation |  |  |  |  |  |  |  |
| I would want compensation if my employee  became severely ill and had to take additional time off work |  |  |  |  |  |  |  |

Now that you have considered more about human challenge studies, to what extent do you agree or disagree that a human challenge study with coronavirus should take place in Singapore?

1. Strongly agree
2. Agree
3. Somewhat agree
4. Neutral
5. Somewhat disagree
6. Disagree
7. Strongly disagree

Would you like to explain your answer and/or make any final comments about anything that’s been covered in this survey?

_________________________________________________________________________

__________________________________________________________________________________________________________________________________________________

**Section B. Exploring public practices and attitudes towards payment in challenge study**

The following survey is exploring practices and attitudes towards payment in Challenge Studies. Challenge Studies are studies in which participants are purposely infected with disease, often in order to help develop a vaccination. Because the infection will challenge the body’s immune system, they are called Challenge Studies. Like most clinical trials with healthy volunteers, participants in these studies usually receive money or paid compensation for their involvement in the Challenge Study.

First, you will read about two real and ongoing Challenge Studies.

Please indicate whether you think people should be allowed to participate in these studies, and if so how much the participants should be paid in each of the studies.

Please read carefully through this scenario.

| Dengue is a virus that is passed to people by the bite from an infected mosquito. Currently there is no treatment for dengue fever, and available vaccines are not sufficiently safe or effective.  To this end, a group of researchers is running a Challenge Study that involves infecting humans with a weakened dengue strain. This strain has previously been tested in a human challenge study and shown to result in only a mild illness. The study procedure is as follows: participants are infected with dengue through an injection. Participants then attend the clinic daily until they develop signs of dengue fever, and depending on the severity of these symptoms may be admitted to an inpatient facility (e.g. a hospital) until resolution. After resolution participants continue to be regularly followed up in the clinic for 28 days after infection. Throughout the study, participants will have blood and urine tests.  The researchers expect that participants will experience mild symptoms of a fever and rash and may also have headache, backache and nausea. Any other complications are very unlikely. |
| --- |

Do you believe people should be allowed to participate in this study?

1. Yes

2. No

[Display if No] In your opinion, why people should NOT be allowed to participate in this study?

1. [free text response]

[Display if Yes] In your opinion, how much should people be paid for their involvement in this study?

As a reference point:

- The National Wages Council in Singapore has recommended a “basic wage threshold” of SGD1400 in guidelines issued in May 2021. This translates to approximately 8 SGD / hour.
- The median gross monthly income of a full time employed resident in Singapore in 2021 is $4680. This translates to approximately 25 SGD / hour.

1. No money should be offered for participating in the study
2. Less than or equal to 4 SGD/hour
3. 6 SGD/hour
4. 8 SGD/hour
5. 10 SGD/hour
6. 12.5 SGD/hour
7. 15 SGD/hour
8. 17.5 SGD/hour
9. 20 SGD/hour
10. 22.5 SGD/hour
11. 25 SGD/hour
12. 27.5 SGD/hour
13. More than or equal to 30 SGD/hour

| The COVID-19 pandemic has caused many deaths worldwide and has been one of the biggest global health problems we have faced. Vaccines and treatments are now available to treat COVID-19, but these are imperfect and are limited by the emergence of new variants.  To this end, researchers are running a Challenge Study in which participants are infected with the SARS-CoV-2 coronavirus through an inoculation in the nose. Only participants who have previously been vaccinated against COVID-19 will be eligible to join the study. The study procedure is as follows: participants will be admitted to an isolation room at a hospital and infected with SARS-CoV-2. They will remain an inpatient and under observation for the next 14 days, until they are confirmed to be no longer infectious. After discharge from the hospital there will be continued followed up at an outpatient clinic with regular visits initially weekly and then every 4 months for one year. Throughout the study, participants will have blood and urine tests and throat swabs.  Participants are expected to experience symptoms of COVID-19, such as sore throat, cough, fever, and headache. Antiviral medication will be available if needed, but it is unlikely that any complications will occur. |
| --- |

Do you believe people should be allowed to participate in this study?

1. Yes
2. No

[Display if No] If your opinion, why people should NOT be allowed to participate in this study?

1. [free text response]

[Display if Yes]In your opinion, how much should people be paid for their involvement in this study?

As a reference point:

- The National Wages Council in Singapore has recommended a “basic wage threshold” of SGD1400 in guidelines issued in May 2021. This translates to approximately 8 SGD / hour.
- The median gross monthly income of a full time employed resident in Singapore in 2021 is $4680. This translates to approximately 25 SGD / hour.

1. No money should be offered for participating in the study
2. Less than or equal to 4 SGD/hour (less than 1344 SGD for 14 days)
3. 6 SGD/hour (2016 SGD for 14 days)
4. 8 SGD/hour (2688 SGD for 14 days)
5. 10 SGD/hour (3360 SGD for 14 days)
6. 12.5 SGD/hour (4200 SGD for 14 days)
7. 15 SGD/hour (5040 SGD for 14 days)
8. 17.5 SGD/hour (5880 SGD for 14 days)
9. 20 SGD/hour (8160 SGD for 14 days)
10. 22.5 SGD/hour (7560 SGD for 14 days)
11. 25 SGD/hour (8400 SGD for 14 days)
12. 27.5 SGD/hour (9240 SGD for 14 days)
13. More than or equal to 30 SGD/hour (10080 SGD for 14 days)

The following questions will ask you about what factors you believe should be taken into account when determining payment for Challenge Studies participants.

| Jesse is interested in becoming a research participant in a study where they would be infected with a disease X and required to stay at a medical centre for 10 days. There is a 70% chance that they will experience a mild, treatable illness such as a flu-like or diarrhoeal illness, and there is a 1 in 100,000 (one in one hundred thousand) chance that they will experience any other more serious side effects, such as inflammation of the heart or liver that would likely require a hospital admission and further treatment and could potentially be fatal in a small proportion of cases. This is similar to the chance of experiencing a serious allergic reaction to an mRNA COVID-19 vaccine. |
| --- |

Please rank the following factors in the order of importance when determining the payment for Jesse. Please put the factor you consider the most important first, followed by the second most important factor and so forth.

1. Time required in trial
2. Pain involved in trial
3. Risk of serious side effects
4. Inconvenience involved in trial
5. Number of non-invasive investigations involved in trial (for example urine sample, saliva swab, ultrasound)
6. Number of invasive investigations involved (for example blood tests, investigations requiring sedation)

Are there any factors which you think should not be taken into account when determining the payment for Jesse?

1. Time required in trial
2. Pain involved in trial
3. Risk of serious side effects
4. Inconvenience involved in trial
5. Number of non-invasive investigations involved in trial (for example urine sample, saliva swab, ultrasound)
6. Number of invasive investigations involved (for example blood tests, investigations requiring sedation)
7. No, all the above factors should be taken into account

You will move on to the next set of questions. These questions ask you to indicate how much you agree with statements about how payment should be determined in Challenge Studies.

Please bear in mind that there is no “right” or “wrong” answer – all we are interested in is your intuitive response.

Please Indicate how much you agree with the following statements:

|  | Strongly disagree | Disagree | Somewhat disagree | Neither agree nor disagree | Somewhat agree | Agree | Strongly Agree |
| --- | --- | --- | --- | --- | --- | --- | --- |
| Research participants should never be paid or reimbursed for their involvement in a research study |  |  |  |  |  |  |  |
| Research participants should be paid for their involvement in a research study. Payment should aim to only reimburse the participant for time and travel expenses |  |  |  |  |  |  |  |
| Research participants should be paid for their involvement in a research study. The hourly rate of payment should be determined by what other unskilled labourers are paid but no extra money should be given for the risks involved |  |  |  |  |  |  |  |
| Research participants should be paid for their involvement in a research study. The base hourly rate of payment should be determined by what other unskilled labourers are paid with extra money given dependent on the risks involved |  |  |  |  |  |  |  |
| Research participants should be paid for their involvement in a research study. Payment should be determined by the research investigators and funders and it should depend on the current market demands (the demand/supply of participants and how quickly recruitment needs to occur) |  |  |  |  |  |  |  |
| It is not concerning to give large amounts of money to Challenge Study participants. It is just like getting paid to perform other jobs with physical risks involved, such as being a firefighter or working as a window cleaner on a high rise building |  |  |  |  |  |  |  |
| Offering large amounts of money to become a Challenge Study participant could make participants feel like they must participate in this research |  |  |  |  |  |  |  |

|  | Strongly disagree | Disagree | Somewhat disagree | Neither agree nor disagree | Somewhat agree | Agree | Strongly Agree |
| --- | --- | --- | --- | --- | --- | --- | --- |
| Offering large amounts of money to participants for being involved in Challenge Studies could mean that some people will freely choose to become involved because the benefits of participating (including the value of the money to them) outweigh the risks |  |  |  |  |  |  |  |
| Offering large amounts of money to participants for being involved in Challenge Studies could impair their judgement. This means they may choose to become involved in the research despite it not being in their best interests |  |  |  |  |  |  |  |
| It is ethically permissible to allow someone to participate in a Challenge Study for no money but solely because of the benefits the results of a study could provide to their community |  |  |  |  |  |  |  |
| If it is ethically permissible to allow someone to participate in a Challenge Study for no money, but because of the benefits the results of a study could provide to their community, then it is also ethically permissible for researchers to offer whatever amount of money is necessary to recruit the required amount of participants for the study |  |  |  |  |  |  |  |

You are now on the final part of this study. Please answer these short demographic questions.

**Section C. Background questions**

The following section asks questions about your background and your current circumstances. This is important because we want to capture views from across the population living in Singapore, not just one area of society.

What is your gender?

1. Male
2. Female
3. Other

What is your age in years?

1. [box to enter response]

What is your residential status?

1. Citizen
2. Permanent resident
3. Employment/Work pass holder
4. Other, specify

Which of the following best describes your ethnic group?

1. Chinese
2. Malay
3. Indian
4. Eurasian
5. Other, specify:

What is your highest educational or work-related qualification?

1. No formal qualifications
2. Primary school
3. Secondary school
4. Post-secondary
5. Diploma
6. Degree/Post-graduate degree

What is your current employment status?

1. Employed full time
2. Employed part time
3. Self-employed
4. Unemployed
5. Student
6. Retired
7. Homemaker
8. Other – please specify

Do you work in the healthcare sector?

1. Yes
2. No

What is your marital status?

1. Married
2. Single
3. Divorced
4. Widowed

What is your total annual income (the total amount of money you make in a year before taxes and other deductibles are taken from it) that includes salary, overtime pay, tips, bonuses or any commissions?

1. Under SGD 20000 per year
2. SGD 20000 - 60000 per year
3. SGD 60001 - 10000 per year
4. Above SGD 100000 per year
5. Prefer not to say

Do you have caring responsibilities for any child(ren) and/or other dependents?

1. Yes, I do
2. No, I don’t

In which study did you participate in this research clinic?

1. PRIBIVAC
2. VAP3
3. RVAC
4. Other
5. Not applicable

How many times had you participated in similar clinical research?

1. [box to enter response]

- The End -

THANKS for your participation in this survey!
